# R2R3-MYB Transcription Factor *PlMYB108* Confers Drought Tolerance in Herbaceous Peony (*Paeonia lactiflora* Pall.)

**DOI:** 10.3390/ijms222111884

**Published:** 2021-11-02

**Authors:** Yanqing Wu, Tingting Li, Zhuoya Cheng, Daqiu Zhao, Jun Tao

**Affiliations:** 1 Joint International Research Laboratory of Agriculture and Agri-Product Safety, The Ministry of Education of China, Institutes of Agricultural Science and Technology Development, Yangzhou University, Yangzhou 225009, China; yqwu@yzu.edu.cn; 2 College of Horticulture and Plant Protection, Yangzhou University, Yangzhou 225009, China; tingtingliyzu@163.com (T.L.); zhuoya.edu@outlook.com (Z.C.); dqzhao@yzu.edu.cn (D.Z.)

**Keywords:** MYB, drought stress, flavonoid, ROS, *Paeonia lactiflora*

## Abstract

The MYB transcription factor (TF) is crucial for plant growth, development, and response to abiotic stress, but it is rarely reported in the herbaceous peony (*Paeonia lactiflora* Pall.). Here, an MYB TF gene was isolated, and based on our prior mRNA data from *P. lactiflora* samples, it was treated with drought stress (DS). Its complete cDNA structure was 1314 bp, which encoded 291 amino acids (aa). Furthermore, using sequence alignment analysis, we demonstrated that *PlMYB108* was an R2R3-MYB TF. We also revealed that PlMYB108 was primarily localized in the nucleus. Its levels rose during DS, and it was positively correlated with drought tolerance (DT) in *P. lactiflora*. In addition, when *PlMYB108* was overexpressed in tobacco plants, the flavonoid content, antioxidant enzyme activities, and photosynthesis were markedly elevated. Hence, the transgenic plants had stronger DT with a higher leaf water content and lower H_2_O_2_ accumulation compared to the wild-type (WT) plants. Based on these results, *PlMYB108* is a vital gene that serves to increase flavonoid accumulation, reactive oxygen species (ROS), scavenging capacity, and photosynthesis to confer DT. The results would provide a genetic resource for molecular breeding to enhance plant DT.

## 1. Introduction

As the global climate continues to warm, a large quantity of water evaporates, resulting in a massive reduction in soil water content and subsequent drought conditions [1]. Being a major abiotic stressor, drought severely affects growth, development [2], yield [3], and quality [4] of plants, which in turn negatively affects agricultural supply. In China, the northern regions are mostly dry or semidry. As a result, in these areas, drought remains a major factor restricting landscaping construction. Therefore, the cultivation of drought-tolerant plant varieties and the generation of a low water consumption model and an esthetic plant configuration model are the main directions of green construction in the northern region.

Plants have evolved a variety of survival mechanisms in order to adapt to arid environments. One such mechanism is the regulation of drought-resistant genes [5]. Drought-resistant genes are mainly divided into two types: functional and regulatory. Transcription factors (TFs) belong to regulatory genes, which modulate the levels of multiple functional genes related to drought resistance. Multiple studies revealed that this is the most effective approach to enhancing plant drought resistance [6]. At present, the TF families associated with plant drought resistance mainly include bZIP (Basic leucine zipper), AP2/ERF (APETALA2/Ethylene Response Element Binding Factors), MYB (myeloblastoma), WRKY, NAC, etc. [7,8,9]. Of this list, MYB is the most prevalent in plants [10]. Its *N*-terminus has a highly conserved MYB DNA-binding domain, and it encodes proteins with 51–53 amino acid (aa) [11]. The MYB TFs are further categorized into four groups, namely, 1R-MYB/MYB-related, R2R3-MYB, 3R-MYB (R1R2R3-MYB), and 4R-MYB based on the number of unit repetitions within the MYB domain [12,13]. Among them, the R2R3-MYB TFs contribute greatly to plant growth and development [14] and abiotic stress response [15,16]. In a study by Zhang et al. [17], it was reported that the wheat gene R2R3-MYB TF *TaMYB30-B* improved drought stress (DS) tolerance in transgenic *Arabidopsis*. Similarly, the poplar R2R3-MYB TF *PtrMYB94* modulates abscisic acid signaling to enhance drought tolerance (DT) in plants [18]. These studies indicate that the R2R3-MYB TF can be used as an important target for improving drought resistance in plants.

The herbaceous peony (*Paeonia lactiflora* Pall.) is a perennial herbaceous flower that can survive for long periods of time and can massively reduce landscaping costs. *P. lactiflora* has a long history of cultivation in China, and it is mainly cultivated in the northern regions. In these regions, however, drought is a major stress factor affecting the cultivation of *P. lactiflora*. In *P. lactiflora*, the physiological and biochemical responses under drought stress were investigated [19], and drought stress changed the expression of differentially expressed genes (DEGs) involved in the reactive oxygen species (ROS) system, chlorophyll degradation and photosynthetic capacity, secondary pathways of biosynthesis, and sugar metabolism [20]. However, studies involving the TFs that regulate *P. lactiflora* DT are rarely reported. Herein, an R2R3-MYB TF *PlMYB108* was extracted from *P. lactiflora*, and its function in conferring DT was examined in transgenic tobacco plants.

## 2. Results

### 2.1. Extraction and Sequence Analysis of PlMYB108

**Figure 1:**
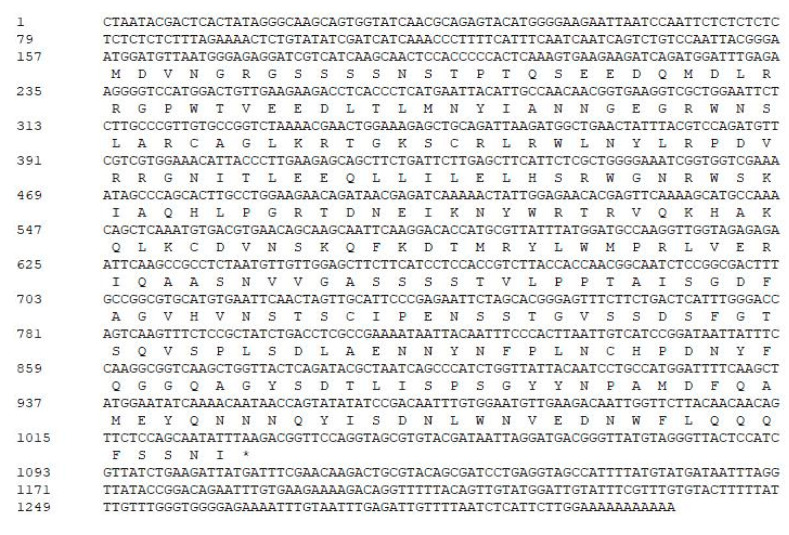
cDNA sequence of *PlMYB108* and its deduced amino acid sequence. The deduced amino acid sequence is shown underneath the corresponding nucleotide sequence, the others are noncoding regions, and the stop code was indicated with *.

Based on our previous transcriptome data of *P. lactiflora* treated with drought stress, (SAR: SRP131648) [20], the TF *MYB108* (Unigene0031519), produced the largest difference from the controls; we examined its full-length cDNA via RACE. In addition, the result demonstrates that *MYB108* has an open reading frame (ORF) of 876 bp, a 5′ UTR (untranslated region) of 156 bp and a 3′ UTR of 282 bp. Moreover, it encodes a 291 aa protein (Figure 1).

Using the web software ProtParam, we further revealed that PlMYB108 has a molecular weight of 33,009.52 Da and a theoretical isoelectric point (pI) of 6.22. Based on this evidence, we identified this protein to be both unstable and hydrophilic. Moreover, using the web softwares TMHMM and SignalP 5.0, we estimated that PlMYB108 had no signal peptide and transmembrane sites. This suggests that PlMYB108 is likely a nonsecretory and/or nontransmembrane protein.

**Figure 2:**
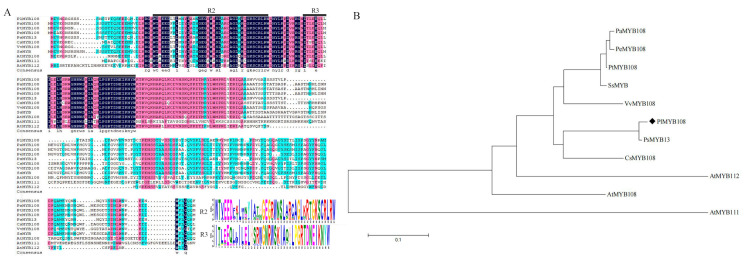
Sequences alignment and phylogenetic tree of PlMYB108 and seven other MYB proteins from plants. (**A**) Sequences alignment. The R2 and R3 MYB domains shown refer to two repeats of the MYB DNA-binding domain of MYB proteins. (**B**) Phylogenetic tree. Protein accession numbers are as follows: *Populus alba* MYB108 (XP_034907007), *Populus trichocarpa* MYB108 (XP_002316060), *Populus euphratica* MYB108 (XP_011010946), *Paeonia suffruticosa* MYB13 (QIG55696), *Camellia sinensis* MYB108 (XP_028115735), *Vitis vinifera* MYB108 (RVW26470), *Salix suchowensis* MYB (KAG5237142), *Arabidopsis thaliana* MYB108 (NP_187301), *Arabidopsis thaliana* MYB111 (NP_199744), and *Arabidopsis thaliana* MYB112 (NP_564519).

The homologous sequence alignments of PlMYB108 with *Populus alba* MYB108 (XP_034907007), *Populus trichocarpa* MYB108 (XP_002316060), *Populus euphratica* MYB108 (XP_011010946), *Paeonia suffruticosa* MYB13 (QIG55696), *Camellia sinensis* MYB108 (XP_028115735), *Vitis vinifera* MYB108 (RVW26470), *Salix suchowensis* MYB (KAG5237142), *Arabidopsis thaliana* MYB108 (NP_187301), *Arabidopsis thaliana* MYB111 (NP_199744), and *Arabidopsis thaliana* MYB112 (NP_564519) were performed by DNAMAN5.2.2, and they shared a homology rate of 62.39%. Based on a sequence analysis, PlMYB108 was shown to contain both *N*-terminal R2 and R3 MYB DNA-binding domains, thereby suggesting that it is an R2R3-MYB TF (Figure 2A). Meanwhile, we also generated a phylogenetic tree via the Neighbor-Joining method (NJ) and MEGA5.05. Additionally, we followed numerous protein sequence alignments using ClustalW. Together, the data demonstrate that PlMYB108 is strongly correlated with PsMYB13 (QIG55696) (Figure 2B).

### 2.2. PlMYB108 Expression Pattern Analysis in P. lactiflora Exposed to DS

**Figure 3:**
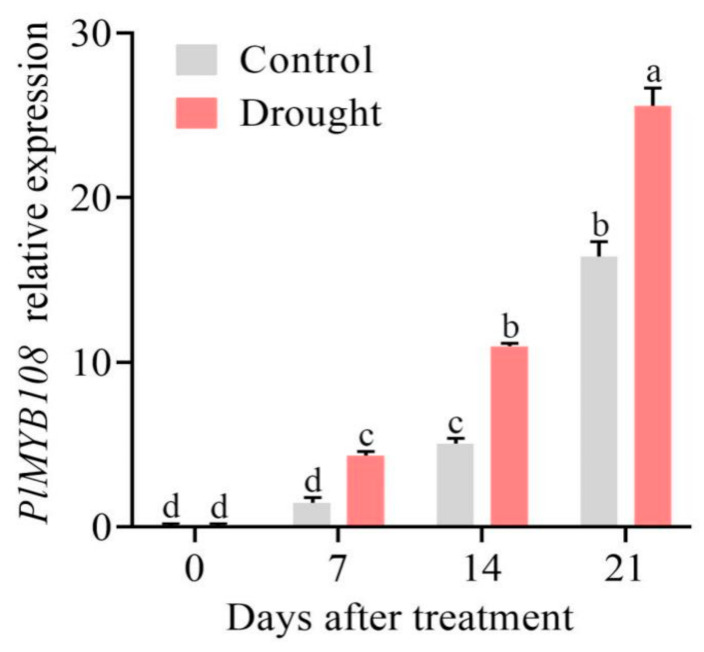
Relative expression levels of *PlMYB108* in *P. lactiflora* under drought stress. The values represent the mean ± SD, and different letters indicate significant differences according to Duncan’s multiple range test (*p* < 0.05).

The *PlMYB108* expression pattern was determined in *P. lactiflora* exposed to DS (Figure 3). With increasing duration, the *PlMYB108* levels increased remarkably in controls, whereas under natural DS, the *PlMYB108* levels still increased, reaching their highest expression on the 21st day post exposure. Relative to the controls, the *PlMYB108* levels in *P. lactiflora* exposed to DS were markedly high.

### 2.3. Examining Localization of the PlMYB108 Protein

**Figure 4:**
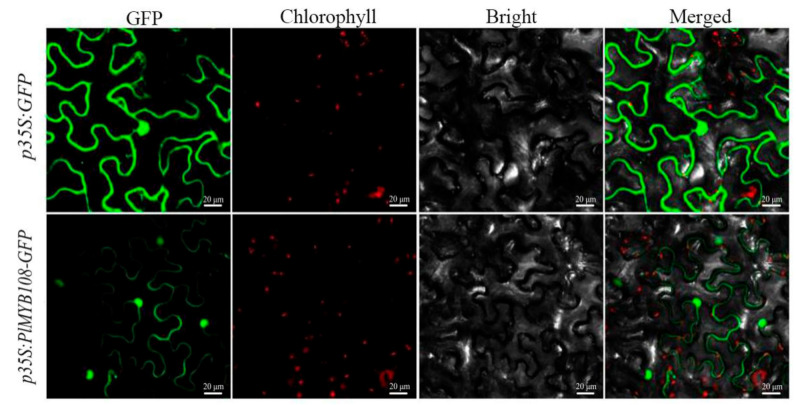
Subcellular localization of PlMYB108 in tobacco leaves. Note: From left to right are superimposed photos of GFP (green fluorescent protein), chloroplast autofluorescence, bright field, and three channels of target gene/empty vector, respectively.

To examine the localization of the PlMYB108 protein, the constructed plasmid *p35S:PlMYB108*-*GFP* was transformed in tobacco leaves. Based on our data, the emitted fluorescence is strongly expressed in the nucleus, which indicates that PlMYB108 is primarily a nuclear protein (Figure 4).

### 2.4. PlMYB108 Conferred DS Tolerance in Tobacco

**Figure 5:**
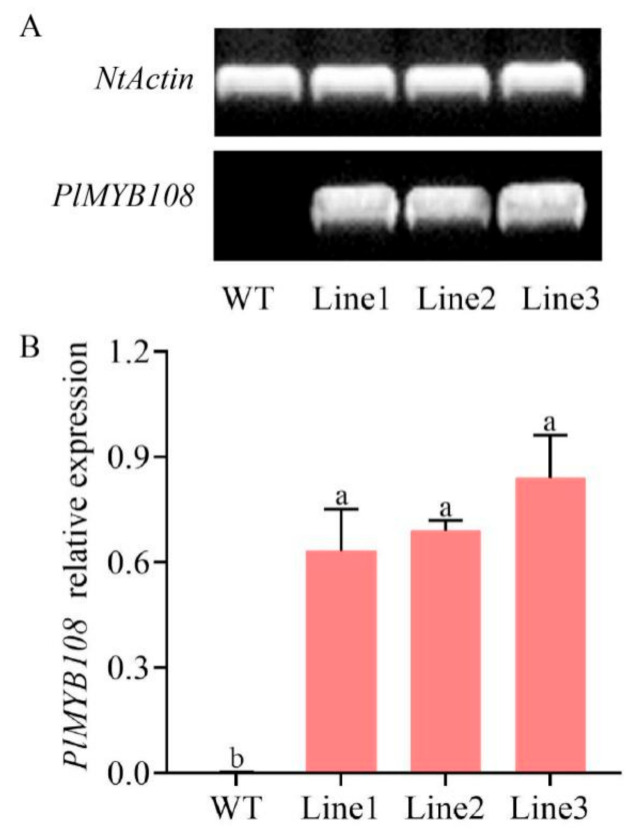
Identification of transgenic tobacco lines. (**A**) PCR analysis of *PlMYB108* mRNA; (**B**) Relative expression level of *PlMYB108* in leaves using qRT-PCR; the values represent the mean ± SD, and different letters indicate significant differences according to Duncan’s multiple range test (*p* < 0.05).

PCR and qRT-PCR were used for the identification of transgenic tobacco plants in this study. After 2 months of cultivation, the leaves of all the tobacco plants were employed as materials for identification. The PCR analysis revealed that all the tobacco plants produced a single bright band of *NtActin*, and the products of *PlMYB108* were detected only in the transgenic lines (TL) but not in WT (Figure 5A). In addition, we performed a further analysis using qRT-PCR. We demonstrated that the *PlMYB108* levels in TL were markedly elevated relative to WT (Figure 5B). These results suggest that *PlMYB108* was successfully transformed in tobacco plants.

**Figure 6:**
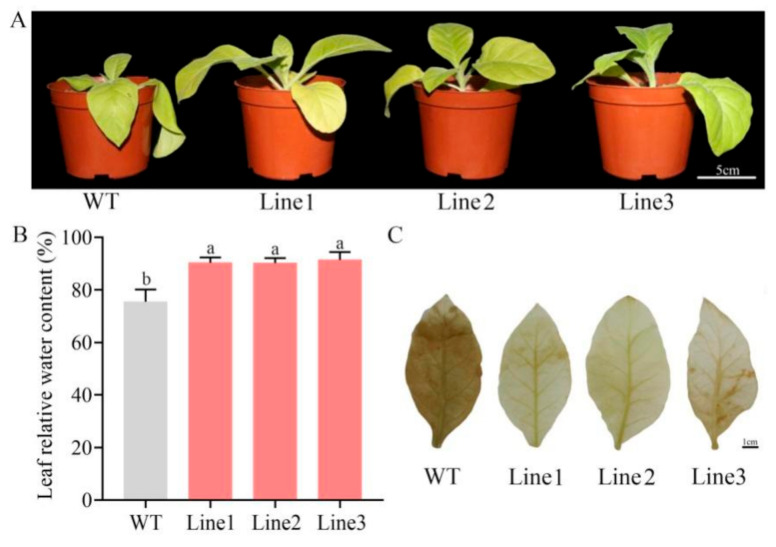
Phenotype and stress physiological indices of WT and transgenic lines under drought stress. (**A**) Phenotype of WT and transgenic lines under drought stress; (**B**) Relative leaf water content; (**C**) H_2_O_2_ accumulation was detected by DAB staining. Tobacco plants were stressed by drought for 10 days. The values represent the mean ± SD, and different letters indicate significant differences according to Duncan’s multiple range test (*p* < 0.05).

Next, the tobacco plants were exposed to natural DS for 10 days. Based on our results, the leaves of WT and TL showed massive differences. The WT leaves exhibited obvious wilting and drooping, whereas the TL ones exhibited sustained growth (Figure 6A). We also evaluated the relative leaf water content (RLWC) and H_2_O_2_ accumulation to assess the growth status of the TL post DS. Based on our results, the TL exhibited markedly increased RLWC and lower H_2_O_2_ accumulation when compared to the WT (Figure 6B,C).

### 2.5. PlMYB108 Increased Flavonoid Accumulation and Antioxidant Enzyme Activities

**Figure 7:**
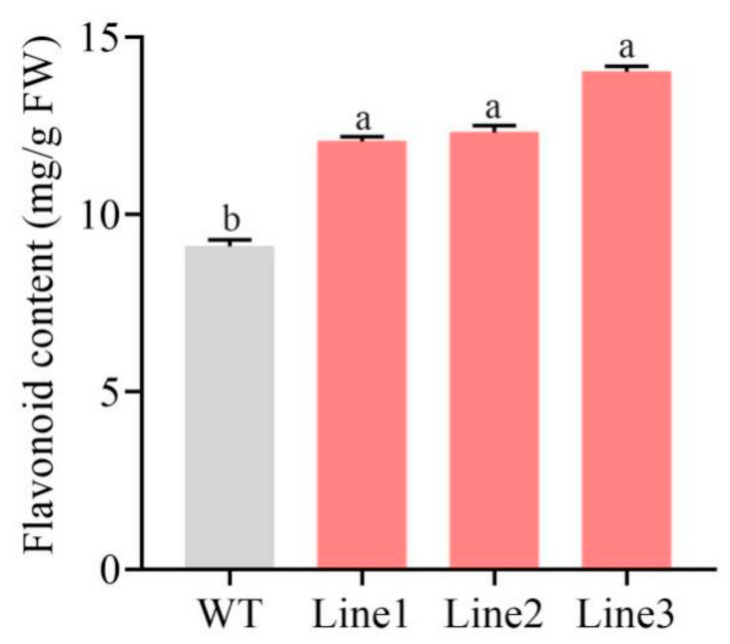
Flavonoid content of WT and transgenic lines under drought stress. Tobacco plants were stressed by drought for 10 days. The values represent the mean ± SD, and different letters indicate significant differences according to Duncan’s multiple range test (*p* < 0.05).

**Figure 8:**
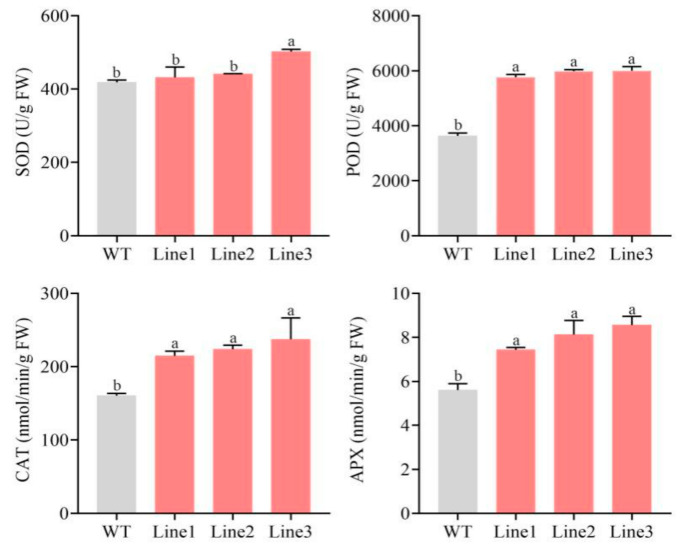
Antioxidant enzyme activities of WT and transgenic lines under drought stress. Tobacco plants were stressed by drought for 10 days. The values represent the mean ± SD, and different letters indicate significant differences according to Duncan’s multiple range test (*p* < 0.05).

To clarify how *PlMYB108* confers DS tolerance within tobacco plants, the flavonoid content and antioxidant enzyme activities were assessed. As shown in Figure 7, the flavonoid content was markedly elevated (~40.52%) in TL compared to WT. Moreover, the antioxidant enzyme activities, including superoxide dismutase (SOD, EC 1.15.1.1), peroxidase (POD, EC 1.11.1.7), catalase (CAT, EC 1.11.1.6), and ascorbate peroxidase (APX, EC 1.11.1.11) were markedly increased, with values that were respectively 1.09, 1.63, 1.40, and 1.43 times that of WT (Figure 8).

### 2.6. PlMYB108 Enhanced Photosynthesis

**Figure 9:**
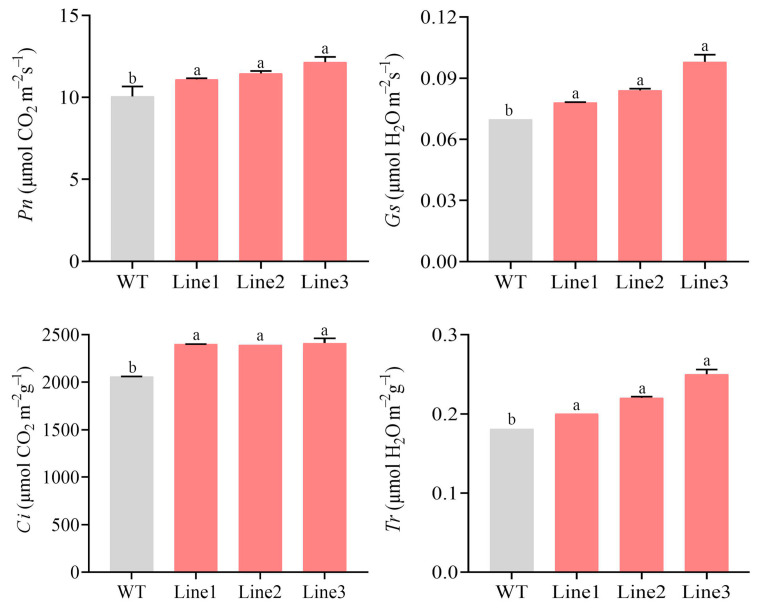
Photosynthetic characteristics of WT and transgenic lines under drought stress. Tobacco plants were stressed by drought for 10 days. The values represent the mean ± SD, and different letters indicate significant differences according to Duncan’s multiple range test (*p* < 0.05).

**Figure 10:**
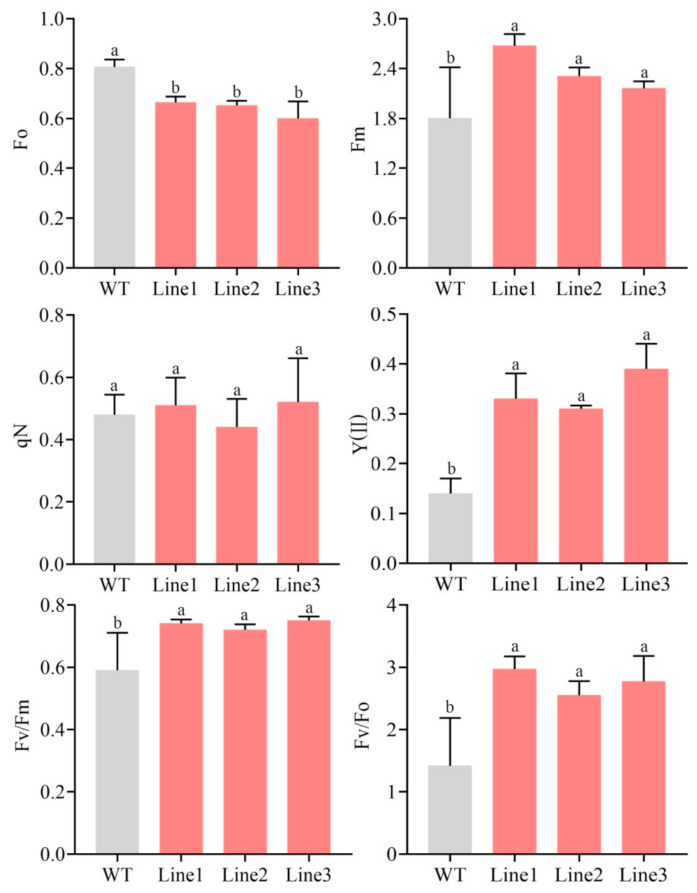
Chlorophyll fluorescence parameters of WT and transgenic lines under drought stress. Tobacco plants were stressed by drought for 10 days. The values represent the mean ± SD, and different letters indicate significant differences according to Duncan’s multiple range test (*p* < 0.05).

Subsequently, the photosynthetic characteristics, including net photosynthesis rate (*P*n), stomatal conduction (*G*s), intercellular CO_2_ concentration (*C*i), and transpiration rate (*T*r) were analyzed. Compared to WT, *P*n, *G*s, *C*i, and *T*r increased by 14.98%, 12.44%, 16.56%, and 24.06% in TL, respectively (Figure 9). Additionally, the chlorophyll fluorescence parameters, including the minimum fluorescence (Fo), the maximum fluorescence (Fm), the non-photochemical quenching (qN), the actual photosynthetic efficiency of light system II (Y(II)), the variable fluorescence to maximum fluorescence ratio (Fv/Fm), and the variable fluorescence to minimum fluorescence (Fv/Fo) ratio, were also measured. Compared to WT, Fm, Y(II), Fv/Fm, and Fv/Fo were all remarkably elevated in TL compared to WT. Moreover, qN was slightly higher, while Fo was significantly lower (Figure 10), in TL than in WT.

## 3. Discussion

Water shortage is a major problem facing the world today, and many garden flowers cannot develop under drought conditions. Therefore, cultivating drought-tolerant varieties is of great significance for landscaping in arid conditions. Multiple factors modulate plant drought resistance. When plants are drought-stressed, they resist and adapt to drought in different ways [21]. However, TFs that modulate the expression of genes related to plant resistance and stress have gained much attention [6]. MYB is among the highly prevalent TF families in plants [10]. Among them, the R2R3-MYB subtype is widely expressed and contributes greatly to plant growth and development [14] and response to abiotic stress [15,16]. At present, the R2R3-MYB TFs have been cloned in numerous plants. For example, Wang et al. [22] isolated a strawberry R2R3-MYB TF *FvMYB24* that possessed the full-length coding region and encoded an estimated protein of 284 aa. Similarly, Zhang et al. [23] isolated *PmMYBa1* from *Prunus mume*, which carried a conserved R2R3 MYB domain and belonged to the anthocyanin-associated subgroup 6 of the R2R3-MYB family. In this study, the full-length *PlMYB108* cDNA was retrieved via RACE technology, which encoded a protein of 291 aa. Furthermore, using sequence analysis, we demonstrated that PlMYB108 carried both the N-terminal R2 and R3 MYB DNA-binding domains. This suggests that *PlMYB108* is an R2R3-MYB TF with a high homology with *MYB* from other plants, as reported in a prior study [24].

In recent years, there have been extensive studies on the function of *MYB* in plant abiotic stress response. For example, Qin et al. [25] found that the expression of *TaMYB33* in wheat leaves was induced by NaCl, PEG, and ABA treatments. Likewise, Wu et al. [26] also showed that PEG6000, NaCl, and ABA treatments upregulated *ZmMYB3R* levels in maize. These results suggest that *TaMYB33* and *ZmMYB3R* might modulate plant stress response. Here, we detected *PlMYB108* levels using qRT-PCR. Under natural DS, *PlMYB108* levels were markedly upregulated in *P. lactiflora*, which might positively correlate with DT in *P. lactiflora*. Subsequently, we investigated the subcellular localization of PlMYB108 and found that it primarily resided in the nucleus, which mirrors the RhMYB108 study in rose plants [27]. Based on this evidence, PlMYB108 is a nuclear protein, which might serve as a TF that plays a regulatory role in the nucleus.

A previous study reported cloning *TaPIMPI*, with *MYB*-typical characteristics, based on the homologous sequence of *Arabidopsis AtMYB108*. Furthermore, its overexpression in wheat markedly enhanced drought resistance in plants [28]. To further confirm the DT activity of *PlMYB108* in *P. lactiflora*, *PlMYB108* was incorporated into a model tobacco plant, as evidenced by PCR and qRT-PCR. Next, the tobacco plants underwent natural DS for 10 days. Following this, the WT leaves showed significant wilting and drooping, whereas the TL remained normal in its development. This phenotypic difference strongly suggests that *PlMYB108* confers DT in tobacco. Moreover, relative leaf water content and H_2_O_2_ accumulation were markedly elevated in the TL compared to WT, which revealed that *PlMYB108* can effectively maintain a normal water requirement and remove ROS.

Flavonoids represent a class of secondary metabolites that exert either positive or negative effects on plant growth. Nakabayashi et al. reported that overexpressing *AtMYB12* and *AtMYB75* in *Arabidopsis* remarkably enhanced DT via the overaccumulation of flavonoids [29]. In *P. lactiflora*, DS significantly increased flavonoid accumulation [20]. Moreover, in our study, the flavonoid content in TL was markedly increased (40.52%) compared to WT. This, in turn, exerted positive effects on the DS of tobacco plants. In addition, antioxidant enzyme activities in TL were significantly elevated in our study relative to WT, which, in turn, increased the ROS scavenging capacity and reduced H_2_O_2_ accumulation. 

Photosynthesis provides both energy and material resources for plants. In this study, *P*n, *G*s, *C*i, and *T*r were markedly increased in TL versus WT. This suggests that *PlMYB108* alleviates the DS-mediated suppression of photosynthesis, which is in accordance with results from the *P. ostii* caffeoyl-CoA *O*-methyltransferase overexpression examinations [30]. In addition, chlorophyll fluorescence parameters are used as internal indices of plants’ adaptation to the eco-environment, and their changes during environmental stress can be used to judge the degree of damage to photosynthetic organs. In this study, Fm, Y(ΙΙ), Fv/Fm, and Fv/Fo in TL were markedly elevated, relative to WT, and the value of Fv/Fm was approximately 0.75, suggesting that DS caused little damage to the PSΙΙ reaction center in TL compared to WT. Therefore, the TLs were better at maintaining normal photosynthesis during DS. Our conclusions would provide a theoretical basis for future studies attempting to enhance DS tolerance in *P. lactiflora* via genetic engineering.

## 4. Materials and Methods

### 4.1. Extraction and Bioinformatic Analysis of PlMYB108

Total *P. lactiflora* RNA isolation was done with a MiniBEST Plant RNA Extraction Kit (TaKaRa, Kyoto, Japan). The entire cDNA was retrieved via rapid-amplification of cDNA ends (RACE) using a 3′ full RACE Core Set v2.0 (TaKaRa, Kyoto, Japan) and a SMARTer^TM^ RACE cDNA Amplification Kit (Clontech, Mountain View, CA, USA) with targeted primers (Table S1). The PCR product was then separated on an agarose gel, excised, and sent for sequencing.

The aa composition, protein molecular weight, pI, stability, and hydropathicity were estimated via the ProtParam (http://web.expasy.org/protparam/, accessed on 20 August 2021) software. The transmembrane domain was analyzed via the TMHMM server 2.0 software (http://www.cbs.dtu.dk/services/TMHMM, accessed on 20 August 2021). The signal peptide was estimated via the SignalP 5.0 Server (http://www.cbs.dtu.dk/services/SignalP/, accessed on 20 August 2021). The homologous PlMYB108 sequence alignment with other species was done via DNAMAN5.2.2, and the phylogenetic tree was generated via NJ of MEGA5.05.

### 4.2. Expression Pattern Analysis of PlMYB108 P. Lactiflora Exposed to DS

Five-year-old *P. lactiflora* plants in potting soil (loam:peat:perlite, 1:1:1) were employed for the DS-stimulated *PlMYB108* expression pattern analysis. The *P. lactiflora* plants were placed in a glass greenhouse on the Wenhui Road campus of Yangzhou University. They were separated into 2 groups, which each contained six plants: one group received water at 17:00 daily and served as the control group, and the other group received natural DS. The top leaves were collected at 0, 7, 14, and 21 days post exposure for an assessment of the *PlMYB108* levels, using qRT-PCR via the BIO-RAD CFX Connect^TM^ Optics Module (Bio-Rad, Hercules, CA, USA). All values were computed via the 2^−∆∆Ct^ relative threshold cycle (Ct) method [31]. Primer sequences are summarized in Table S2.

### 4.3. Subcellular PlMYB108 Localization 

This analysis was done using tobacco leaves and confocal laser microscopy (Nikon C2-ER, Tokyo, Japan). The *p35S:PlMYB108*-*GFP* gene fusions were synthesized to retrieve the *PlMYB108* product (Figure S1). The ORF of *PlMYB108* was amplified using primers carrying *BsmB* I restriction sites (forward 5′-CAGTGGTCTCACAACATGGATGTTAATGGGAGAGG-3′, reverse 5′-CAGTGGTCTCATACAAATATTGCTGGAGAACTGTT-3′), which were then digested and introduced to the expression vectors with T4 DNA ligase (TaKaRa, Kyoto, Japan) to produce a set of *p35S:PlMYB108*-*GFP* fusions, which were then sequenced for further validation. Subsequent transformations of the *p35S:PlMYB108*-*GFP* and the empty *p35S:GFP* vector were conducted as reported previously [32].

### 4.4. PlMYB108 Overexpression in TL Tobacco

The *p35S-PlMYB108* plasmid was incorporated into the *Agrobacterium tumefaciens* strain *EHA105* using the freeze–thaw procedure. In turn, the strains were incorporated into tobacco (*Nicotiana tabacum* ‘k326′) via the leaf disc transformation procedure. Plasmid transformation was then verified using PCR and qRT-PCR. The primers used are summarized in Table S3. 

### 4.5. DS Exposure Affecting PlMYB108 in TL Tobacco

The TL and WT tobacco plants were maintained for 2 months prior to use in natural DS experiments. The TL tobacco plants contained three lines, each line contained six plants. The plants underwent DS (i.e., no water) for 10 days. Next, the photosynthetic characteristic and chlorophyll fluorescence parameters were measured, and essential physiological variables such as RLWC, H_2_O_2_, flavonoid content, and antioxidant enzyme activities were assessed. Following this, the leaves were kept at −80 °C with liquid nitrogen until further gene expression analyses.

### 4.6. Measurements of Physiological Indexes, Photosynthetic Characteristics, and Chlorophyll Fluorescence Parameters

An oven (Shanghai Jinghong Laboratory Instrument Co., Ltd., Shanghai, China) and balance (Suzhou Science Instrument Co., Ltd., Suzhou, China) were used to evaluate the RLWC. In brief, the leaf weight was measured and documented as fresh weight (FW). Next, the leaves were heated to 105 °C for 5 min in an oven, then cooled to 65 °C for > 2 h. The dried leaf weight was weighed again and recorded as dry weight (DW). Lastly, the RLWC was computed as follows: (FW−DW)/FW × 100%.

Diaminobenzidine (DAB) staining was employed for the detection of hydrogen peroxide (H_2_O_2_) accumulation. In short, leaves were submerged in 0.1 mg/mL DAB in 50 mM Tris-acetate buffer (pH 5.0) in the dark at 25 °C. Following a 24 h incubation, the samples were removed and boiled in 95% (*v/v*) ethanol for ≥15 min, before capturing images with a camera (Canon 50D, Tokyo, Japan).

The flavonoid content and antioxidant enzyme activities, namely SOD, POD, CAT, and APX, were assessed with reagent kits (Suzhou Corning Biotechnology Co., Ltd., Suzhou, China). Finally, a portable photosynthesis system (Li-Cor LI-6400, Nebraska, USA) and a chlorophyll fluorescence spectrometer (Heinz Walz GmbH 91090 Effeltrich, Nuremberg, Germany) were employed for the measurement of photosynthetic and chlorophyll fluorescence, respectively.

### 4.7. Statistical Analysis

The data presented are the means of at least three randomized replicates with standard deviations. A variance analysis was done with the SAS/STAT statistical analysis package (v6.12, SAS Institute, Cary, NC, USA).

## 5. Conclusions

*PlMYB108*, a R2R3-MYB TF, was extracted from *P. lactiflora*, and its protein was located in the nucleus. The *PlMYB108* expression was positively correlated with DT in *P. lactiflora*. Based on our analysis, *PlMYB108* is an essential gene that markedly enhances flavonoid accumulation, ROS scavenging capacity, and photosynthesis and eventually confers DT in tobacco. Our conclusion would provide a theoretical basis for future studies attempting to enhance DS tolerance in *P. lactiflora* via genetic engineering.

## Data Availability

Not applicable.

## Supplementary Materials

The following are available online at https://www.mdpi.com/article/10.3390/ijms222111884/s1.

## Abbreviations

MYB	Myeloblastoma
TF	Transcription factor
DS	Drought stress
aa	Amino acids
DT	Drought tolerance
WT	Wild-type
ROS	Reactive oxygen species
bZIP	Basic leucine zipper
ORF	Open reading frame
DEGs	Differential expression genes
RLWC	Relative leaf water content
RACE	Rapid-amplification of cDNA ends
DAB	Diaminobenzidine
qRT-PCR	Quantitative real-time polymerase chain reaction

## References

[1] Farooq M., Wahid A., Kobayashi N., Fujita D., Basra S.M.A. (2009). Plant drought stress: Effects, mechanisms and management. Agron. Sustain. Dev..

[2] He X., Xu L., Pan C., Gong C., Wang Y., Liu X., Yu Y. (2020). Drought resistance of Camellia oleifera under drought stress: Changes in physiology and growth characteristics. PLoS ONE.

[3] Dhansu P., Kulshreshtha N., Kumar R., Raja A.K., Pandey S.K., Goel V., Ram B. (2021). Identification of Drought-Tolerant Co-canes Based on Physiological Traits, Yield Attributes and Drought Tolerance Indices. Sugar Tech.

[4] Ozturk A., Erdem E., Aydin M., Karaoglu M.M. (2021). The effects of drought after anthesis on the grain quality of bread wheat depend on drought severity and drought resistance of the variety. Cereal Res. Commun..

[5] Zhao D.Q., Zhang X.Y., Fang Z.W., Wu Y.Q., Tao J. (2019). Physiological and transcriptomic analysis of tree peony (Paeonia section Moutan DC.) in response to drought stress. Forests.

[6] Ahmed R.F., Irfan M., Shakir H.A., Khan M., Chen L. (2020). Engineering drought tolerance in plants by modification of transcription and signalling factors. Biotechnol. Biotechnol. Equip..

[7] Baldoni E., Genga A., Cominelli E. (2015). Plant MYB Transcription Factors: Their Role in Drought Response Mechanisms. Int. J. Mol. Sci..

[8] Leng P., Zhao J. (2019). Transcription factors as molecular switches to regulate drought adaptation in maize. Theor. Appl. Genet..

[9] Joshi R., Wani S., Singh B., Bohra A., Dar Z., Lone A., Pareek A., Singla-Pareek S.L. (2016). Transcription Factors and Plants Response to Drought Stress: Current Understanding and Future Directions. Front. Plant Sci..

[10] Riechmann J.L., Heard J., Martin G., Reuber L., Jiang C.-Z., Keddie J., Adam L., Pineda O., Ratcliffe O.J., Samaha R.R. (2000). Arabidopsis Transcription Factors: Genome-Wide Comparative Analysis Among Eukaryotes. Science.

[11] Stracke R., Werber M., Weisshaar B. (2001). The R2R3-MYB gene family in Arabidopsis thaliana. Curr. Opin. Plant Biol..

[12] Prouse M.B., Campbell M. (2012). The interaction between MYB proteins and their target DNA binding sites. Biochim. Biophys. Acta (BBA)-Bioenerg..

[13] Bilaud T., Koering C.E., Binet-Brasselet E., Ancelin K., Pollice A., Gasser S.M., Gilson E. (1996). The telobox, a Myb-related telo-meric DNA binding motif found in proteins from yeast, plants and human. Nucleic Acids Res..

[14] Zhou C.G., Li C.H. (2016). A novel R2R3-MYB transcription factor BpMYB106 of birch (Betula platyphylla) confers increased pho-tosynthesis and growth rate through up-regulating photosynthetic gene expression. Front. Plant Sci..

[15] Zhang C.-Y., Liu H.-C., Zhang X.-S., Guo Q.-X., Bian S.-M., Wang J.-Y., Zhai L.-L. (2020). VcMYB4a, an R2R3-MYB transcription factor from Vaccinium corymbosum, negatively regulates salt, drought, and temperature stress. Gene.

[16] Wei Q., Chen R., Wei X., Liu Y., Zhao S., Yin X., Xie T. (2020). Genome-wide identification of R2R3-MYB family in wheat and functional characteristics of the abiotic stress responsive gene TaMYB344. BMC Genom..

[17] Zhang L., Zhao G., Xia C., Jia J., Liu X., Kong X. (2012). A wheat R2R3-MYB gene, TaMYB30-B, improves drought stress tolerance in transgenic Arabidopsis. J. Exp. Bot..

[18] Fang Q., Wang X., Wang H., Tang X., Liu C., Yin H., Ye S., Jiang Y., Duan Y., Luo K. (2019). The poplar R2R3 MYB transcription factor PtrMYB94 coordinates with abscisic acid signaling to improve drought tolerance in plants. Tree Physiol..

[19] Wang Q., Zhao R., Chen Q.H., da Silva J.A.T., Chen L.Q., Yu X.N. (2019). Physiological and biochemical responses of two herbaceous peony cultivars to drought stress. Hortscience.

[20] Li T., Wang R., Zhao D., Tao J. (2020). Effects of drought stress on physiological responses and gene expression changes in herbaceous peony (Paeonia lactiflora Pall.). Plant Signal. Behav..

[21] Yang X.Y., Lu M.Q., Wang Y.F., Wang Y.R., Liu Z.J., Chen S. (2021). Response Mechanism of Plants to Drought Stress. Horticulturae.

[22] Wang S., Shi M., Zhang Y., Xie X., Sun P., Fang C., Zhao J. (2021). FvMYB24, a strawberry R2R3-MYB transcription factor, improved salt stress tolerance in transgenic Arabidopsis. Biochem. Biophys. Res. Commun..

[23] Zhang Q., Hao R., Xu Z., Yang W., Wang J., Cheng T., Pan H., Zhang Q. (2017). Isolation and functional characterization of a R2R3-MYB regulator of Prunus mume anthocyanin biosynthetic pathway. Plant Cell Tissue Organ Cult..

[24] Mandaokar A., Browse J. (2009). MYB108 acts together with MYB24 to regulate jasmonate-mediated stamen maturation in Arabidopsis. J. Plant Physiol..

[25] Qin Y., Wang M., Tian Y., He W., Han L., Xia G. (2012). Over-expression of TaMYB33 encoding a novel wheat MYB transcription factor increases salt and drought tolerance in Arabidopsis. Mol. Biol. Rep..

[26] Wu J., Jiang Y., Liang Y., Chen L., Chen W., Cheng B. (2019). Expression of the maize MYB transcription factor ZmMYB3R enhances drought and salt stress tolerance in transgenic plants. Plant Physiol. Biochem..

[27] Zhang S., Zhao Q.C., Zeng D.X., Xu J.H., Zhou H.G., Wang F.L., Ma N., Li Y.H. (2019). RhMYB108, an R2R3-MYB transcription factor, is involved in ethylene- and JA-induced petal senescence in rose plants. Hortic. Res..

[28] Zhou M.P., Zhou X.Q., Yao J.B., Zhang Z.Y., Zhang P., Yang X.M., Ma H.X. (2013). Preliminary analysis of drought tolerance in MYB transgenic wheat. Jiangsu J. Agr. Sci..

[29] Nakabayashi R., Yonekura-Sakakibara K., Urano K., Suzuki M., Yamada Y., Nishizawa T., Matsuda F., Kojima M., Sa-kakibara H., Shinozaki K. (2014). Enhancement of oxidative and drought tolerance in Arabidopsis by overaccumulation of antioxidant flavonoids. Plant J..

[30] Zhao D.Q., Luan Y.T., Shi W.B., Zhang X.Y., Meng J.S., Tao J. (2021). A Paeonia ostii caffeoyl-CoA O-methyltransferase confers drought stress tolerance by promoting lignin synthesis and ROS scavenging. Plant Sci..

[31] Zhao D., Shi W., Xia X., Tang Y., Tao J. (2019). Microstructural and lignin characteristics in herbaceous peony cultivars with different stem strengths. Postharvest Biol. Technol..

[32] Zhao D.Q., Xia X., Su J.H., Wei M.R., Wu Y.Q., Tao J. (2019). Overexpression of herbaceous peony HSP70 confers high temperature tolerance. BMC Genom..

