# Supplementary material for: R2R3-MYB Transcription Factor PlMYB108 Confers Drought Tolerance in Herbaceous Peony (Paeonia lactiflora Pall.)
Source: Int J Mol Sci. 2021 Nov 2;22(21):11884. doi: 10.3390/ijms222111884 (PMC8584830; doi:10.3390/ijms222111884)
Supplement: Supplementary file 1 [file ijms-22-11884-s001.zip › ijms-1388232-supplementary.pdf]

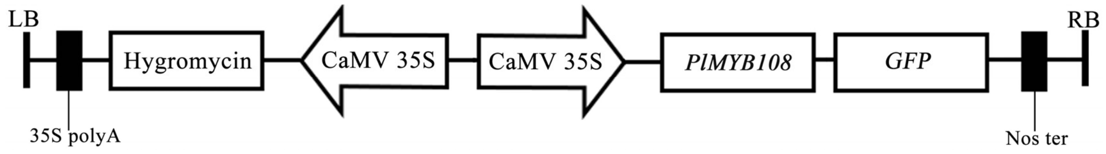

Figure S1. Overexpression of *PlMYB108* promoted by CaMV35s promoter for tobacco transformation.

Table S1

Gene-specific primers used for *PlMYB108* isolation.

Primer	Sequence (5' - 3')	Description
PlMYB108$_1$	AACAACGGTGAAGGTCGC	1st of 3' RACE
PlMYB108$_2$	TTCAAGCCGCCTCTAATG	2nd of 3' RACE
PlMYB108$_3$	CGAGGTCAGATAGCGGAGAAACTTGACTGG	5' RACE (Clontech)

Table S2

Gene-specific primers used in gene expression analysis.

Gene	Species	GenBank accession	Forward primer sequence (5' - 3')	Reverse primer sequence (5' - 3')
Actin	*Paeonia lactiflora*	JN699053	GTTGCCCTTGATTACGAG	GCTTCCATTCCGATTAGTG
MYB108	*Paeonia lactiflora*	-	AGAAGAAACTCCCGTGCTA	GCGTTATTTATGGATGCC

Table S3

Gene-specific primers used for transformed tobacco line identification.

Gene name	Gene ID	Forward primer (5' - 3')	Reverse primer (5' - 3')	Application
NtActin	AB158612	TCCTCATGCAATTCTTCG	ACCTGCCCATCTGGTAAC	PCR and qRT-PCR identification
PlMYB108$_4$	-	CAGTGGTCTCACAACATGGATGTT AATGGGAGAGG	CAGTGGTCTCATACAAATATTGCT GGAGAACTGTT	PCR identification
PlMYB108$_5$	-	AGAAGAAACTCCCGTGCTA	GCGTTATTTATGGATGCC	qRT-PCR identification
